# Synergic effect between 5-fluorouracil and celecoxib on hypoxic gastric cancer cells

**DOI:** 10.3892/mmr.2021.12332

**Published:** 2021-08-02

**Authors:** Xiao-Qian Zhang, Xiu-E Sun, Wen-Dong Liu, Yu-Guang Feng, Hong-Mei Zhang, Li-Hong Shi, Xiu-Ning Sun, Yan-Qing Li, Zhi-Xing Gao

Mol Med Rep 11: 1160-1166, 2014; DOI: 10.3892/mmr.2014.2783

Following the publication of the above article, the authors have realized that Fig. 2 was published with an incorrect data panel: Essentially, Fig. 2D was erroneously selected from the representative images of the Fig. 1C data group during figure compilation.

The authors were able to locate their original data, and the corrected version of Fig. 2, featuring the corrected data panel for Fig. 2D, is shown below. All the authors agree with this Corrigendum, and are grateful to the Editor of *Molecular Medicine Reports* for allowing them to publish it. The authors also regret that this inadvertent error was included in the paper, even though it did not substantially alter any of the major conclusions reported in the study, and apologize to the readership for any inconvenience caused.

## Figures and Tables

**Figure 2.**
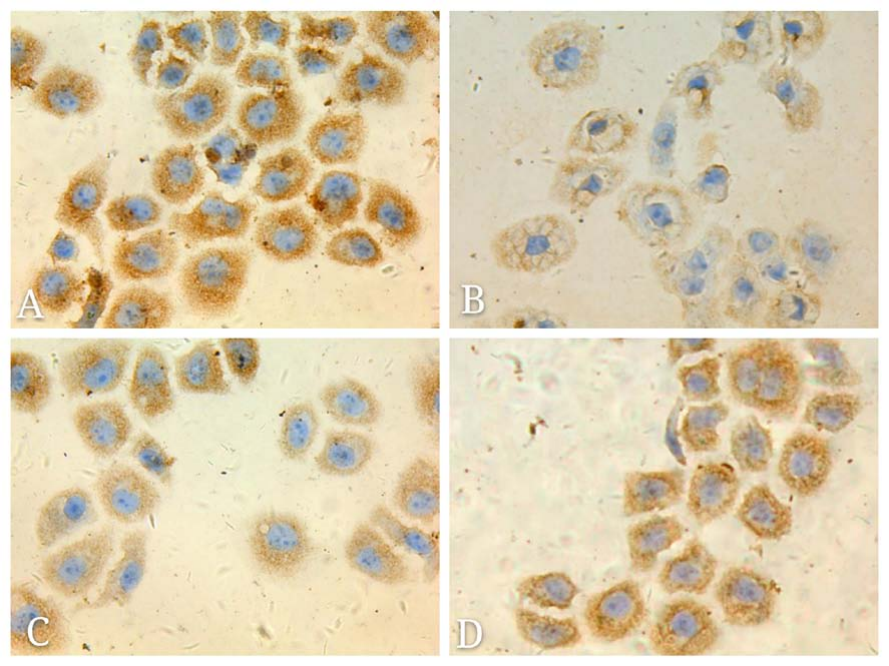
Adenosine triphosphate-binding cassette sub-family G member 2 expression in each group by immunocytochemistry (magnification, ×400). (A) 5-fluorouracil group; (B) celecoxib group; (C) combination group; and (D) hypoxia control group

